# Thermal Stability and Flammability of Styrene-Butadiene Rubber-Based (SBR) Ceramifiable Composites

**DOI:** 10.3390/ma9070604

**Published:** 2016-07-21

**Authors:** Rafał Anyszka, Dariusz M. Bieliński, Zbigniew Pędzich, Przemysław Rybiński, Mateusz Imiela, Mariusz Siciński, Magdalena Zarzecka-Napierała, Tomasz Gozdek, Paweł Rutkowski

**Affiliations:** 1 Institute of Polymer and Dye Technology, Faculty of Chemistry, Lodz University of Technology, Stefanowskiego 12/16, Lódź 90-924, Poland; dariusz.bielinski@p.lodz.pl (D.M.B.); 800655@edu.p.lodz.pl (M.I.); mariusz.sicinski@p.lodz.pl (M.S.); tomasz.gozdek@gmail.com (T.G.); 2 Department of Ceramics and Refractory Materials, Faculty of Materials Science & Ceramics, AGH–University of Science & Technology, Al. Mickiewicza 30, Kraków 30-045, Poland; pedzich@agh.edu.pl (Z.P.); zarzecka@agh.edu.pl (M.Z.-N.); Pawel.Rutkowski@agh.edu.pl (P.R.); 3 Management of Environment Protection and Modeling, The Jan Kochanowski University, Żeromskiego 5, Kielce 25-369, Poland; przemyslaw.rybinski@ujk.edu.pl

**Keywords:** ceramification, ceramization, SBR rubber, composite, thermal stability, flammability, flame retardancy

## Abstract

Ceramifiable styrene-butadiene (SBR)-based composites containing low-softening-point-temperature glassy frit promoting ceramification, precipitated silica, one of four thermally stable refractory fillers (halloysite, calcined kaolin, mica or wollastonite) and a sulfur-based curing system were prepared. Kinetics of vulcanization and basic mechanical properties were analyzed and added as Supplementary Materials. Combustibility of the composites was measured by means of cone calorimetry. Their thermal properties were analyzed by means of thermogravimetry and specific heat capacity determination. Activation energy of thermal decomposition was calculated using the Flynn-Wall-Ozawa method. Finally, compression strength of the composites after ceramification was measured and their micromorphology was studied by scanning electron microscopy. The addition of a ceramification-facilitating system resulted in the lowering of combustibility and significant improvement of the thermal stability of the composites. Moreover, the compression strength of the mineral structure formed after ceramification is considerably high. The most promising refractory fillers for SBR-based ceramifiable composites are mica and halloysite.

## 1. Introduction

Development and utilization of ceramifiable composites have grown significantly since the beginning of the 21st century due to increasing demands originating from fire protection regulations for public property and high-rise buildings. Ceramifiable composites, when exposed to fire or elevated temperature, change their structure from polymer-matrix mineral dispersion into porous and continuous mineral barrier char shield. This specific mineral char not only reduces combustibility of the composites due to the barrier effect but also exhibits mechanical endurance protecting covered objects from external mechanical and thermal stresses.

Ceramifiable composites are key materials for the manufacturing of cables sustaining electrical circuit integrity in case of fire [1], thus providing functioning of essential installations, for example fire sprinklers, fire-proof elevators, camera monitoring, etc. Moreover, they can be used as thermal cover for load-bearing elements of a building [2] or anti-ablative panels for the aerospace industry [3]. The polymer matrix of a ceramifiable composite provides good processability, the ability to ease shaping and it ensures high elasticity. Several phenomena facilitating ceramification of the composites have been identified, namely:

1. Formation of silica bridges between dispersed particles of mineral refractory fillers during thermooxidative decomposition of silicone rubber acting as polymer matrix. Silica is one of the main degradation products of silicone polymers’ decomposition in oxidative atmosphere. In this mechanism the key factor is good adhesion between the silica and thermally stable fillers.
2. Sintering of mineral filler particles through the condensation of hydroxyl groups, which are present on their surface. However, to ensure high effectiveness of this reaction, a high load of mineral fillers is required. On the other hand, based on this mechanism, organic polymers for the matrix can be considered instead of silicones [3,4].
3. Formation of new mineral phases as an effect of chemical reactions between mineral filler particles. The main example of this kind of mechanism is the reaction between calcium oxide and silica, leading to the formation of calcium silicates reinforcing the mineral structure [2,5].
4. Formation of physical connections between particles of thermally stable fillers. This is achieved mainly by the addition of low-softening/melting-point fillers, such as glassy frits with a softening point temperature of 374–525 °C [6,7,8] or boron oxide with a melting point temperature of 450 °C [9,10].
5. Formation of the silicon oxycarbide mineral phase as a result of silicone matrix cross-linking and ceramification. Cross-linking of silicone polymers is one of the mechanisms of their thermal degradation and occurs at very high temperature or under high heating rates [11]. To enhance the cross-linking process efficiency, platinum catalysts [12] or active silica [13] may be added to the silicone matrix.

Recent research in the area of ceramifiable composites has focused on the development of new ceramification-promoting additives. Hu S. et al. manufactured silicone composite filled with ammonium polyphosphate, aluminum hydroxide and mica, which exhibits self-supporting properties during heat treatment up to 1000 °C [14]. In addition, Zhang X. et al. proposed an alternative approach, developing laponite-based pre-organized hollow filler, which physically promotes ceramification when incorporated into silicone rubber matrix [15]. In our recent work we developed silicone rubber–based composites able to create a nano-porous mineral structure during ceremification [16] and composites of considerably high compression strength after ceramification using carbon fibers [17].

Styrene-butadiene rubber (SBR), due to its relatively low cost, good processability, high elasticity, significant abrasion resistance and ease blending with other rubbers, is the most universal and common synthetic rubber. Application of SBR rubber vary from the manufacturing of simple elastic elements, such as gaskets, carpets or tubes, to very sophisticated tire technology. One of the biggest disadvantages of SBR rubber is its high flammability. Therefore, plenty of research projects have been focused on the increase of SBR flame retardancy. A number of them revealed that incorporation of char-forming additives (especially mineral hybrid fillers) into SBR matrix improves its thermal stability and reduces combustibility [18,19]. Ceramification may be interpreted as a developed char formation effect, forming a barrier not only against heat flux and gas compounds (atmospheric oxygen and fuel volatiles produced during thermal degradation of the polymer matrix) but also against external mechanical stress. Thus, SBR rubber could be a promising polymer matrix for ceramifiable composites ensuring required elasticity, and satisfying thermal stability and low flammability in case of fire.

In this paper we are going to introduce styrene-butadiene rubber–based ceramifiable composites to the international scientific community. Combustibility, thermal stability, degradation activation energy and ceramification performance of SBR-based composites containing different mineral refractory fillers are to be discussed.

## 2. Materials and Methods

### 2.1. Materials

Styrene-butadiene rubber synthesized by emulsion method (e-SBR) used as continuous phase for all the composites, trade name KER 1500, was purchased from Synthos S.A., Oswiecim, Poland. The rubber contains 22–25 wt. % of bonded styrene, 5.0–7.5 wt. % of organic acids, max. 0.7 wt. % of volatile matters, max. 0.4 wt. % of soaps and max. 0.4 wt. % of total ash. Its viscosity (ML 1+4; 100 °C) is 45 ÷ 55 °ML. Precipitated silica “Arsil^®^” used as a reinforcing filler was originated from Z. Ch. Rudniki S.A., Oswiecim, Poland. The silica consist of min. 85 wt. % of SiO_2_, min. 7 wt. % of strongly bonded H_2_O. Its 4% water solution exhibits pH between 6.0 and 8.0. Antioxidant (2,2,4-trimethyl-1,2-dihydroquinoline (TMQ)) and cross-linking activators (stearic acid, ZnO), accelerator (*N*-Cyclohexyl-2-benzothiazole sulfenamide (CBS)) and agent (sulfur) were purchased from Torimex-Chemicals Ltd. Sp. z o. o., Konstantynów Łódzki, Poland. Promoting ceramification glass frit “FR-2030” of chemical composition (wt. %): 13.7 Na_2_O, 2.0 BaO, 2.0 Al_2_O_3_, 43.1 SiO_2_, 23.5 ZnO, 15.7 B_2_O_3_ and softening point temperature of 560 °C, was originated from Reimbold & Strick GmbH, Cologne, Germany. Various mineral fillers were used for ceramic layer reinforcement and namely: halloysite “HW” (specific surface area of 60 m^2^/g), produced by PTH Intermark, Gliwice, Poland; calcined kaolin “Polestar 200R” (specific surface area of 8.5 m^2^/g), produced by Imerys Minerals Ltd. (Paris, France); mica (phlogopite) “PW30” (specific surface area of 2.8 m^2^/g), produced by LKAB Minerals GmbH (Lulea, Sweden) Greece and wollastonite “Casiflux FG20 S30.5” (average particle size of *D*_50_ = 10.5 μm), produced by Sibelco Specialty Minerals Europe, Maastricht, The Netherlands. All components were used as received.

### 2.2. Preparation of Rubber Samples

Weight compositions of the samples were identical: 100 phr (weight parts per hundred weight parts of rubber) of rubber, 50 phr of precipitated silica, 105 phr of glass frit, 1 phr of TMQ, 2 phr of CBS, 1 phr of stearic acid, 5 phr of ZnO, 2 phr of sulfur and 145 phr of various mineral fillers, supporting creation of ceramic layer. The difference between the samples was based on the type of the mineral filler added. Samples were designated accordingly to the type of the filler: halloysite—SBR_hal, calcined kaolin—SBR_kao, mica—SBR_mic and wollastonite—SBR_wol. To highlight changes caused by incorporation of a system facilitating ceramification (glass frit, precipitated silica and additional mineral filler) a pristine sample, containing only a curing system and an antioxidant, was prepared and designated as SBR_pris.

All the samples were prepared and formed into flat sheets by two roll (diameter—150 mm; length—200 mm) laboratory mill (Bridge, UK), operating with the friction of 1.1 and rotational speed of the faster roll of 20 rpm (revolutions per minute) and slower of 18 rpm. Kinetics of vulcanization of the prepared mixes were tested using a WG-02 vulcameter (Metalchem, Gliwice, Poland), acc. To PN-ISO 3417:1994. Accordingly to obtained results (Table S1) the mixes were formed and vulcanized into desirable shapes with steel moulds by laboratory press at 160 °C and 10 MPa of pressure, during the optimum time determined vulcametrically.

### 2.3. Experimental Techniques

Mechanical properties of the vulcanizates were tested by means of a Zwick/Roell 1435 static testing machine (stress at different degree of elongation (SE100, SE200 and SE300), tensile strength (*Ts*), tear resistance (*Tes*) and elongation at break (*Eb*)) and a Zwick/Roell hardness testers (Shore hardness, scale *A* and *D*), (Table S2).

Abrasion resistance of the ceramifiable composites was determined by means of Schopper-Schlobach tester (Table S2). Surface of the composites before and after an abrasion test, were evaluated using an optical microscope Leica MZ6, (Figure S1).

Combustibility of the vulcanizates was examined by means of a cone calorimeter manufactured by Fire Testing Technology Ltd. (East Grinstead, UK). Square shaped vulcanizates (100 × 100 mm^2^) ± 1 mm^2^) of 2.0 ± 0.5 mm thickness were placed horizontally to heating source of 35 kW/m^2^. During the test following parameters were collected: heat release rate (HRR), total heat released (THR), averaged heat release rate (ARHE) and mass loss.

Specific heat capacity of the ceramifiable samples was measured using a Netzh LFA-427 device in temperature ranging from 30 to 240 °C.

Thermal stability (TG) of the vulcanizates was measured by means of a Netzh TG-209 thermogravimeter with heating rate of 1, 3 and 5 K/min. The measurements were made under nitrogen atmosphere with gas flow rate of 16 mL/min, whereas the samples mass varied from 7.0 to 16.2 mg.

Ceramification of the vulcanizates was performed in a laboratory furnace where five cylindrical samples (height—8 mm, diameter—16 mm) of each composite were heated up from room temperature up to 950 °C during 2 h. Afterwards the samples were tested for their compression strength in diameter direction by means of a Zwick/Roell Z 2.5 device. Micromorphology of ceramified residues obtained after heat treatment was analyzed by means of Hitachi S-4700 (Tokyo, Japan) scanning electron microscope.

### 2.4. Determination of Activation Energy of Thermal Decomposition by Flynn-Wall-Ozawa Method

A simple model of thermal decomposition of polymer materials can be expressed as conversion: A_solid_ → B_solid_ + C_volatiles_. Where A_solid_ is a starting material undergoing decomposition into other solid (B_solid_) and volatile (C_volatile_) products. Using thermogravimetric analysis, the degree of conversion (thermal decomposition) can be calculated with the following Equation (1) [20].

(1) $$\alpha =\frac{W_{0}-W_{t}}{W_{0}-W_{f}}$$

where α is a degree of conversion, *W*_0_ is a initial mass of the sample, *W*_t_ is current mass of the sample and *W*_f_ is a final mass of the sample.

The Flynn-Wall-Ozawa method is an iso-conversional method allowing estimation of activation energy of decomposition from empiric data [21,22,23], for example from thermogravimetric analysis. At constant conversion (decomposition) degree (α), the plot consisting of logβ (β—heating rate) versus 1/*T* made of the data from several measurements at different heating rates should representing give a straight line which slope indicates the activation energy of decomposition, using the following Equation (2).

(2) $$slope=\frac{d\left(\text{log}\beta \right)}{d\left(\frac{1}{T}\right)}=0.4567\frac{E}{R}$$

where *E* is the activation energy and *R* is the gas constant (8.314 J/(mol·K)).

To apply this model heating rates of 1, 3 and 5 K/min were chosen and measurement were made for conversion rates of 0.3, 0.4, 0.5, 0.6, 0.7 and 0.8.

## 3. Results and Discussion

### 3.1. Combustibility

**Figure 1:**
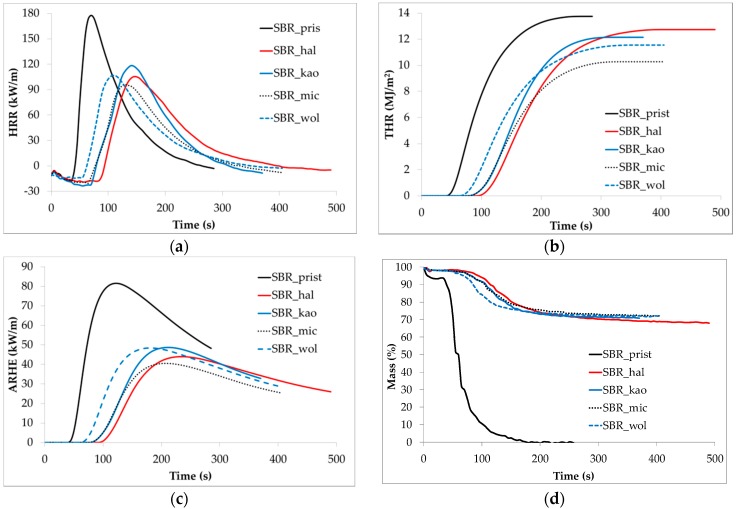
Cone calorimetry analysis of the vulcanizates: heat release rate (**a**); total heat released (**b**); averaged heat release rate (**c**) and mass loss (**d**).

Results obtained a from cone calorimeter show clearly that the addition of ceramification-promoting components decreases the combustibility of SBR rubber (Figure 1 and Table 1). A large amount of thermally stable minerals combined with a glassy frit performing ceramification increases flame retardancy of the composites, due to the barrier effect. The glassy frit particles soften and stick together with particles of thermally stable additives (halloysite, calcined kaolin, mica, wollastonite, silica and zinc oxide), leading to the formation of a continuous barrier structure. Moreover, the presence of a very high amount of mineral fillers of relatively high specific surface area may promote carbonization of SBR rubber and formation of organic char supporting the barrier effect. SBR rubber itself exhibits the ability to carbonize due to a high amount of aromatic rings in its styrene component; moreover, the presence of mineral fillers might intensify this phenomenon. Mineral-carbon char produced on the surface of vulcanizates prevents the bulk of the material from being exposed to oxygen and heat and keeps flammable volatiles inside the composite, which makes its flammability decrease. Ceramifiable composites are generally designed for being able to produce a mineral structure of high strength and tightness, which exhibits significantly higher barrier properties than common char-forming additives. This results in low combustibility of the ceramifiable composites even without the addition of supplementary flame retardants of different acting mechanisms (decreasing the amount of free radicals in the burning zone, diluting the flammable products of a polymer matrix degradation, dissipation of heat from the fire to the bulk of material, etc.). The combustibility parameters vary for the composites due to properties of the additional refractory filler, and its specific surface area, shape, ability to disperse evenly and be distributed in the SBR matrix, and specific interactions with polymer macromolecules.

The lowest heat emission (*HRR* and *ARHE* peaks, *THR*) during the cone calorimetry test is exhibited by the sample filled with mica. This mineral has already proved itself as an effective flame-retardant filler for ceramifiable composites based on silicone rubber due to its surface and thermal properties, its possibly high infrared reflectivity and its flake-like shape with a high area/thickness aspect ratio [24]. The addition of halloysite resulted in a significant increase in the start time of combustion, which is one of the most important flammability parameters; however, the amount of heat generated during the test (*THR*) is also the highest from all of the composites studied. Probably the large specific surface of halloysite and its tubular shape promote the adsorption of combustible products of SBR matrix degradation, delaying the inflammation. Afterwards, during heat increase, the combustible products desorb and burn, increasing the total heat release (*THR*). The kinetics of mass loss during the test is very similar for the all composites except the vulcanizate with wollastonite, which started to burn the earliest.

From the parameters listed in Table 1, the most important from the point of view of combustibility and fire spreading are: the time to ignition (*t*_i_) and peak of heat release rate (*HRR*_p_). Therefore, their ratio could be an interesting factor for the evaluation of materials’ flammability. Taking into account these three parameters, we may suggest that both mica (phlogopite) and halloysite fillers are the most promising fillers for increasing the flame retardancy of SBR-based ceramifiable composites. Moreover, the mechanisms of their flame-retardant properties seem to be slightly different. The addition of halloysite increases the time to ignition value whereas the incorporation of mica decreases the *HRR* peak the most significantly. This may suggest the possibility of obtaining a synergistic effect if these fillers would be added simultaneously into a SBR-based ceramifiable composite. 

**Figure 2:**
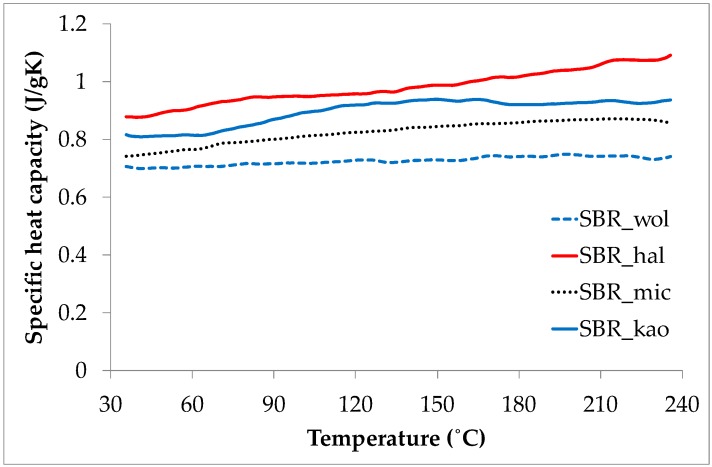
Changes to specific heat capacity of the ceramifiable composites with the increase of temperature.

The most significant increase of the time to ignition for the composite containing halloysite may be explained, regardless of the ceramic-carbon char formation, by four other factors. Firstly, this composite exhibits the highest value of specific heat capacity (Figure 2). Secondly, the activation energy of its thermal decomposition is higher than for the pristine sample (Table 2). Thirdly, its tubular shape and large specific surface promotes the adsorption of combustible volatiles produced from polymer matrix thermal degradation, preventing them from diffusion into the burning zone. Fourthly, the amount of residue after both calorimetry (Table 1) and thermogravimetry (Table 3) tests is the lowest for this composite in comparison to the other composites studied. This suggests that the halloysite powder may contain the highest amount of water, which suppresses the temperature when evaporating. Each composite contains 74.2 wt. % of non-flammable and thermally stable mineral components. Thus, the difference between this value and the value obtained after thermogravimetric analysis is likely due to the amount of water present in the minerals. For example, precipitated silica often contains up to 8 wt. % of water as a result of the method of its preparation.

### 3.2. Thermal Decomposition and Ceramification

**Figure 3:**
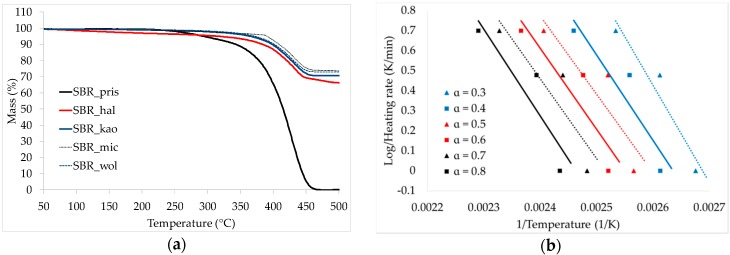

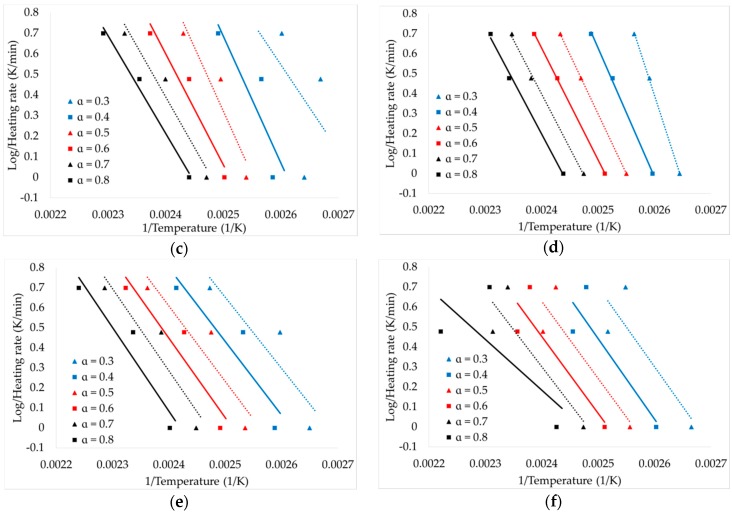
Thermogravimetric characteristic of the samples at heating rate of 5 K/min (**a**) and plots exhibiting Flynn-Wall-Ozawa approach toward estimation of decomposition energy for: SBR_pris (**b**); SBR_hal (**c**); SBR_kao (**d**); SBR_mic (**e**) and SBR_wol (**f**) vulcanizates.

The addition of mineral components promoting ceramification increases the thermal stability of SBR rubber (Figure 3a, Table 3). The beginning of the thermal decomposition of the composites is shifted significantly to higher temperatures, especially for the vulcanizate filled with mica for which the *T*_05_ value is almost 100 °C higher than for the pristine sample, despite the fact that the temperature of the highest decomposition rate for all of the vulcanizates is very similar. Solid residue remaining after thermogravimetric analysis is very high due to the high load of mineral fillers; however, it is lower than calculated value of 74.2 wt. %, probably due to the considerable amount of moisture evaporating during the test. The lowest amount of residue (8 wt. % lower than the calculated value) was determined for the composite filled with halloysite.

The ceramifiable composites studied are complex materials containing lots of components, which may influence thermal decomposition mechanisms very significantly. The active surface of mineral particles may cause a chain scission reaction leading to the acceleration of the decomposition rate as well as carbonization facilitating its deceleration. Rubber macromolecules might adsorb on the porous, large specific surface of active mineral fillers, creating an interphase of the so-called bound rubber of higher thermal stability. Thus, the incorporation of certain fillers may change the activation energy of the thermal decomposition of the composite material. Composites containing halloysite and calcined kaolin exhibit a higher value of activation energy, whereas composites filled with mica and wollastonite exhibit a lower value of activation energy than pristine sample. This shows that for such complex materials, there is no direct and simple relationship between the value of the activation energy of the decomposition and thermal properties or combustibility. 

Surprisingly, the composite filled with calcined kaolin exhibited very high accuracy with the Flynn-Wall-Ozawa model. This suggest that regardless of the heating rate applied (1, 3 or 5 K/min), either there is one predominant mechanism of thermal decomposition or the ratio of the mechanisms involved remains constant.

**Figure 4:**
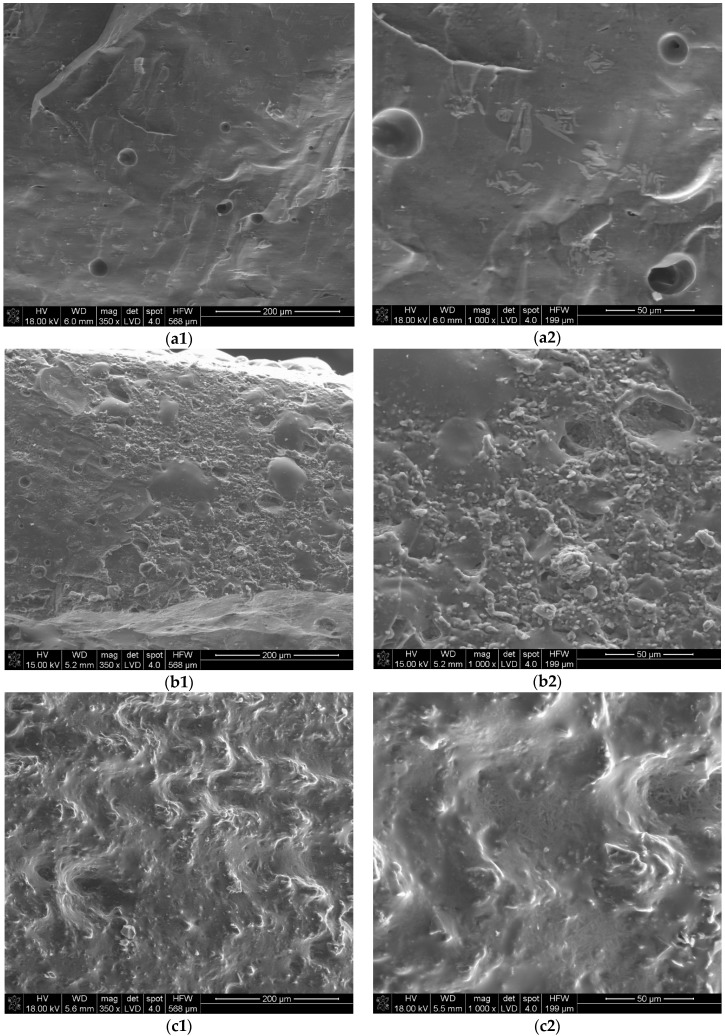

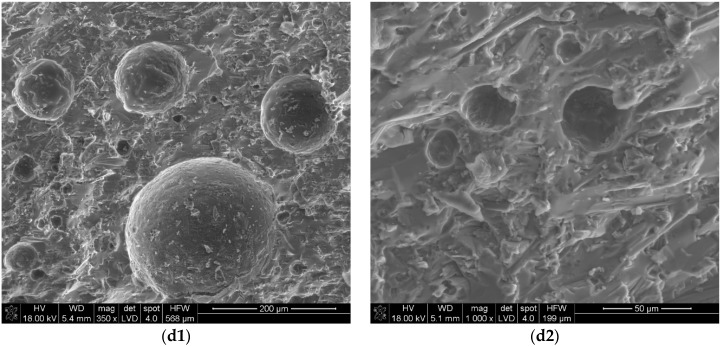
SEM photographs of the ceramified composites’ cross-sections taken under different magnifications of 350× (**a1**, **b1**, **c1** and **d1**) and 1000× (**a2**, **b2**, **c2** and **d2**) for: SBR_hal (**a**); SBR_kao (**b**); SBR_mic (**c**) and SBR_wol (**d**).

The compression strength of the composites after ceramification is very high for these type of materials, especially in comparison to silicone rubber–based composites [17,25]. This could be explained on the basis of their morphology, which depends mostly on the interactions between the frit and mineral fillers (Figure 4). The highest compression strength is exhibited by the composite filled with halloysite, which produced the most homogenous and dense structure after ceramification. The lowest compression strength is exhibited by the composite containing wollastonite, which interacts poorly with the frit. In the SEM pictures, wollastonite needles are visibly separated from the frit, which forms large glassy spheres.

## 4. Conclusions

Ceramifiable elastic composites based on styrene-butadiene rubber were successfully developed. The composites exhibit acceptable processing and mechanical properties. The application of ceramification-promoting fillers noticeably decreased the combustibility and significantly increased the thermal stability of SBR-based composites even though the calculated activation energies of the thermal decomposition were sometimes lower than for the pristine sample. The most promising mineral fillers from the point of view of lowering combustibility, increasing thermal stability and providing high compression strength after ceramification are mica (phlogopite) and halloysite. Taking into account the economic aspect, SBR rubber–based ceramifiable composites may soon become a competitor for silicone rubber–based composites.

## Figures and Tables

**Table 1:** Flammability parameters: time to ignition (*t*_i_), time to flameout (*t*_o_), total heat release (*THR*), mass loss (*m*_l_), heat release rate peak (*HRR*_p_) and its mean value (*HRR*_m_), effective heat of combustion peak (*EHC*_p_) and its mean value (*EHC*_m_), mass loss rate peak (*MLR*_p_) and its mean value (*MLR*_m_), and *HRR*_p_/*t*_i_ ratio.

Parameter	Sample Description				
	SBR_pris	SBR_hal	SBR_kao	SBR_mic	SBR_wol
*t*_i_ (s)	34	82	67	61	51
*t*_o_ (s)	217	399	289	323	304
*THR* (MJ/m^2^)	13.5	12.7	12.1	10.3	11.4
*m*_l_ (%)	100.0	30.9	28.6	27.3	27.4
*HRR*_p_ (kW/m)	177.6	105.3	118.4	95.8	106.9
*HRR*_m_ (kW/m)	72.8	40.2	54.4	39.0	45.5
*EHC*_p_ (MJ/kg)	78.2	66.0	67.4	54.8	79.7
*EHC*_m_ (MJ/kg)	15.0	10.8	11.9	11.5	14.6
*MLR*_p_ (g/s)	0.316	0.138	0.142	0.144	0.145
*MLR*_m_ (g/s)	0.043	0.033	0.040	0.030	0.028
*HRR*_p_/*t*_i_ (kW/ms)	5.22	1.28	1.77	1.57	2.10

**Table 2:** Activation energy of thermal decomposition of the vulcanizates at each conversion rate.

Conversion Rate ɑ	Activation Energy of Thermal Decomposition (kJ/mol)				
	SBR_pris	SBR_hal	SBR_kao	SBR_mic	SBR_wol
0.3	88.4	76.5	158.3	64.7	72.3
0.4	77.6	112.3	117.3	66.8	73.3
0.5	71.1	112.5	108.4	68.4	70.3
0.6	73.6	98.1	101.8	72.3	70.1
0.7	72.6	89.5	98.6	74.7	67.2
0.8	78.7	86.1	96.8	76.0	46.2
Mean	77.0 ± 6.3	95.8 ± 14.5	113.5 ± 23.2	70.5 ± 4.5	66.6 ± 10.2

**Table 3:** Thermal stability parameters of the vulcanizates: temperature of the beginning of decomposition (*T*_05_), temperature of the highest decomposition rate (*T*_hr_) and the rate of decomposition at this temperature (*H*_dr_), the amount of the residue at 500 °C (*P*_500_) and the compression strength of the ceramified samples (*C*_s_).

Parameter	Sample Description				
	SBR_pris	SBR_hal	SBR_kao	SBR_mic	SBR_wol
*T*_05_ (°C)	294	315	365	390	371
*T*_hr_ (°C)	436	432	431	431	432
*H*_dr_ (%/min)	−7.44	−2.39	−2.04	−2.04	−1.99
*P*_500_ (%)	0.0	66.2	70.8	73.6	73.0
*C*_s_ (N)	-	1402 ± 638	1297 ± 789	1221 ± 328	602 ± 315

## Supplementary Materials

The following are available online at www.mdpi.com/1996-1944/9/7/604/s1. Figure S1: Photographs of the ceramifiable samples filled with: halloysite before (a) and after (b) the test, calcined kaolin before (c) and after (d) the test, mica before (e) and after (f) the test and wollastonite before (g) and after (h) the test. Table S1: Optimal vulcanization time τ_90_ and scorch time τ_02_ of the mixes studied. Table S2. Mechanical properties of the vulcanizates studied: Shore hardness, scale *A* and *D*, tear resistance (*Tes*), stress at 100% (SE100), 200% (SE200) and 300% (SE300) of elongation, tensile strength (*Ts*), elongation at break (*Eb*) and abrasion (*A*).

## Additional Information

Ceramifiable composites presented in this article are subject of Polish Patent Application No. 411011.

## Abbreviations

The following abbreviations are used in this manuscript:

APP	ammonium polyphosphate
ARHE	averaged heat release rate
CBS	*N*-Cyclohexyl-2-benzothiazole sulfonamide
CF	carbon fibers
EHC	effective heat of combustion
e-SBR	emulsion-synthesized styrene-butadiene rubber
EVA	poly(ethylene-vinyl acetate)
HRR	heat release rate
LOI	limiting oxygen index
MLR	mass loss rate
OMMT	organofilized montmorillonite
phr	weight parts per hundred weight parts of rubber
rpm	revolutions per minute
THR	total heat released
TMQ	2,2,4-trimethyl-1,2-dihydroquinoline
