# Supplementary material for: Thermal Stability and Flammability of Styrene-Butadiene Rubber-Based (SBR) Ceramifiable Composites
Source: Materials (Basel). 2016 Jul 21;9(7):604. doi: 10.3390/ma9070604 (PMC5456863; doi:10.3390/ma9070604)
Supplement: Supplementary file 1 [file materials-09-00604-s001.pdf]

Supplementary Materials: Thermal Stability and Flammability of Styrene-Butadiene Rubber-Based (SBR) Ceramifiable Composites

Rafał Anyszka, Dariusz M. Bieliński, Zbigniew Pędzich, Przemysław Rybiński, Mateusz Imiela, Mariusz Siciński, Magdalena Zarzecka-Napierała, Tomasz Gozdek and Paweł Rutkowski

Table S1. Optimal vulcanization time τ_{90} and scorch time τ_{02} of the mixes studied.

Parameter	Sample Description				
	SBR_pris	SBR_hal	SBR_kao	SBR_mic	SBR_wol
τ_{02} [1]	7 min. 20 s	8 min. 00 s	5 min. 55 s	6 min. 10 s	6 min. 45 s
τ_{90} [2]	15 min. 45 s	21 min. 30 s	13 min. 10 s	11 min. 25 s	11 min. 20 s

[1] Scorch time is a time of 2% increase of torque value during kinetic of vulcanization test; [2] Optimal vulcanization time is a time of 90% increase of torque value during kinetic of vulcanization test.

Table S2. Mechanical properties of the vulcanizates studied: Shore hardness, scale *A* and *D*, tear resistance (*Tes*), stress at 100% (SE100), 200% (SE200) and 300% (SE300) of elongation, tensile strength (*Ts*), elongation at break (*Eb*) and abrasion (*A*).

Parameter	Sample Description				
	SBR_pris	SBR_hal	SBR_kao	SBR_mic	SBR_wol
Hardness (°Sh*A*)	48.5 ± 0.2	87.1 ± 4.2	85.2 ± 0.4	83.0 ± 1.2	85.7 ± 0.6
Hardness (°Sh*D*)	10.0 ± 0.3	26.9 ± 0.1	26.8 ± 0.1	22.4 ± 0.4	24.8 ± 0.5
Tes (N/mm)	0.4 ± 0.2	41.3 ± 3.0	18.2 ± 1.3	26.2 ± 2.6	20.6 ± 1.8
SE100 (MPa)	0.8 ± <0.1	5.9 ± 0.1	3.7 ± 0.1	3.5 ± 0.1	3.5 ± 0.1
SE200 (Mpa)	1.2 ± <0.1	6.5 ± 0.1	4.4 ± 0.1	4.1 ± 0.1	4.1 ± 0.1
SE300 (Mpa)	1.5 ± <0.1	-	4.9 ± 0.1	4.6 ± 0.1	4.5 ± 0.1
Ts (Mpa)	2.3 ± 0.1	6.6 ± 0.2	5.3 ± 0.2	5.6 ± 0.1	5.5 ± 0.3
Eb (%)	536 ± 71	227 ± 27	424 ± 44	463 ± 88	514 ± 52
A (mm^3)	- [1]	0.43 ± 0.06	0.29 ± 0.04	0.51 ± 0.13	0.35 ± 0.05

[1] Abrasion test was impossible to perform due to high elasticity of the pristine sample, which fall out the device grip.

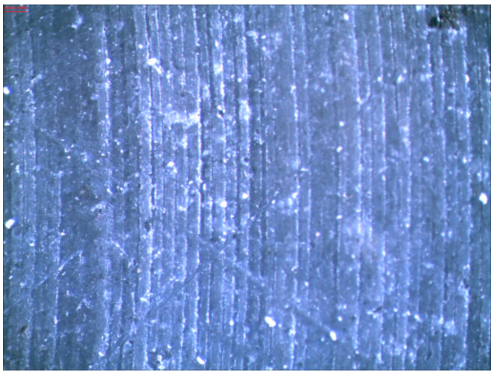

(**a**)

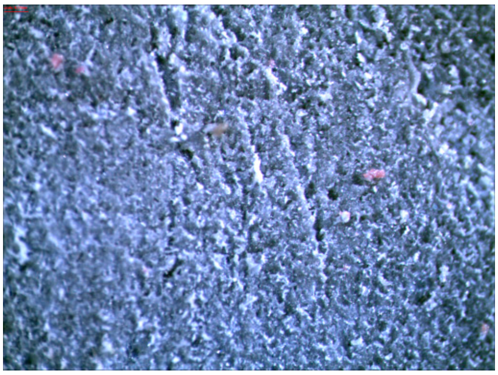

(**b**)

Figure S1. *Cont.*

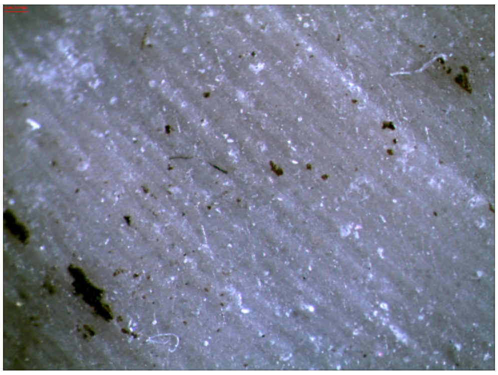

(**c**)

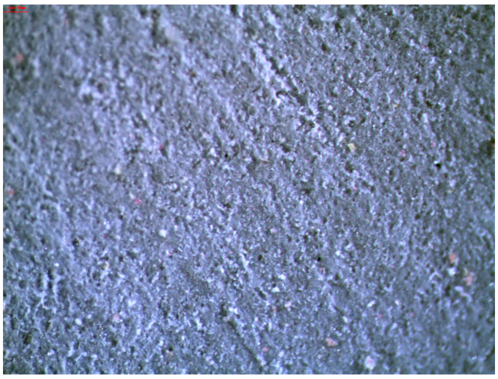

(**d**)

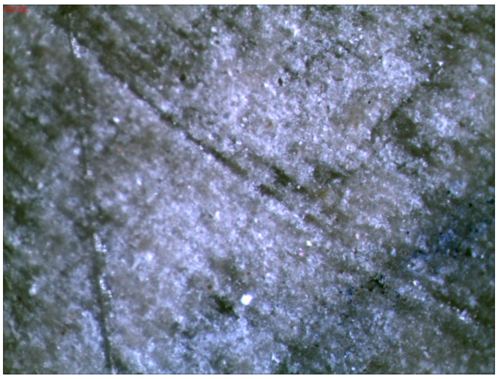

(**e**)

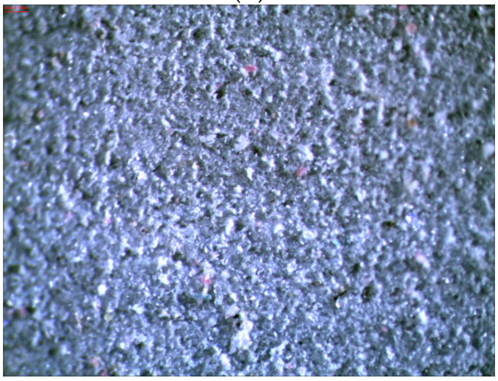

(**f**)

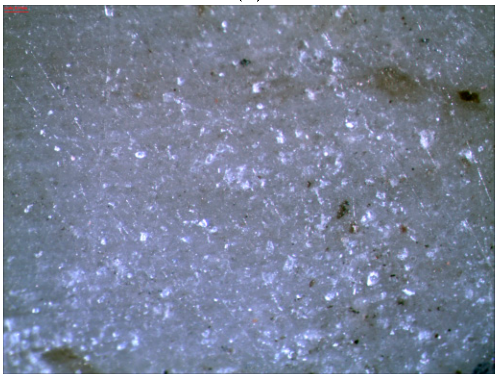

(**g**)

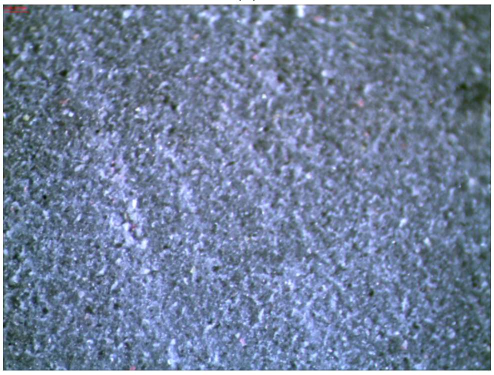

(**h**)

Figure S1. Abrasion test photographs of the ceramifiable samples filled with: halloysite before (**a**) and after (**b**) the test, calcined kaolin before (**c**) and after (**d**) the test, mica before (**e**) and after (**f**) the test and wollastonite before (**g**) and after (**h**) the test.
